# Effects of 25-Hydroxyvitamin D_3_ Combined with Phytase and Probiotic on Calcium–Phosphorus Metabolism, Bone Development, and Growth Performance in Weaned Piglets

**DOI:** 10.3390/nu18091428

**Published:** 2026-04-30

**Authors:** Baoshi Shi, Saiming Gong, Jingjing Wang, Yuyue Xi, Zhiru Tang, Jingchun Gao, Yetong Xu, Zhihong Sun

**Affiliations:** 1 Research Center for Bio-Feed and Molecular Nutrition, College of Animal Science and Technology, Southwest University, Chongqing 400715, Chinam18335761833@163.com (Y.X.);; 2 Key Laboratory of Animal Nutrition and Bio-Feed, Chongqing Municipal Education Commission, Chongqing 400715, China

**Keywords:** weaned piglets, 25-OH-VD_3_, phytase, probiotic, calcium and phosphorus metabolism

## Abstract

**Background/Objectives:** Calcium–phosphorus metabolism is critical for skeletal development in weaned piglets. This study evaluated the effects of dietary 25-hydroxyvitamin D_3_ (25-OH-VD_3_) in combination with phytase and probiotics on mineral metabolism, bone development, and related molecular mechanisms in weaned piglets. **Methods:** Sixty 28-day-old weaned piglets (7.1 ± 1.30 kg) were randomly assigned to four dietary treatments for 31 days (including 3 days of acclimation): CON (basal diet + 50 µg/kg 25-OH-VD_3_), HI (CON + 50 mg/kg phytase), CY (CON +10 mg/kg probiotics), HICY (CON + 50 mg/kg phytase + 10 mg/kg probiotics). Apparent calcium digestibility, serum biochemical indices, bone mineral density (BMD), and mRNA and protein expression of calcium–phosphorus transport- and metabolism-related genes in jejunal mucosa and kidney were assessed. **Results:** Compared with CON, piglets in the HI, CY, and HICY groups showed higher apparent calcium digestibility (*p* < 0.05). Serum transforming growth factor-β was elevated in CY and HICY (*p* < 0.05). HI enhanced metatarsal and toe BMD (*p* < 0.05) and upregulated jejunal solute carrier family 34, member 2 (*SLC34A2*) and *SLC34A3* mRNA expression (*p* < 0.05). In contrast, HICY reduced mRNA expression of transient receptor potential cation channel subfamily V member 6 and calcium-binding protein D28k, as well as of *calcium-binding protein D9k* and *cytochrome P450 27B1* in the kidney (*p* < 0.05). Renal calcium-sensing receptor protein abundance increased in CY (*p* < 0.05). **Conclusions:** Supplementation of 25-OH-VD_3_ with phytase and/or probiotics improved calcium utilization and modulated key transport pathways, contributing to enhanced bone development in weaned piglets. These findings highlight coordinated nutritional regulation of mineral metabolism during early post-weaning growth.

## 1. Introduction

Calcium and phosphorus metabolism and bone development are fundamental biological processes that critically influence growth performance, animal welfare, and production efficiency in pigs. In modern intensive pig production systems, the piglet stage represents a key window for bone development, during which animals are highly susceptible to weaning stress, intestinal dysfunction, and nutrient malabsorption. These challenges often result in impaired calcium and phosphorus utilization, reduced bone mineralization, limb abnormalities, and compromised health status, thereby causing substantial economic losses in the swine industry [1,2]. The association between calcium–phosphorus homeostatic imbalance and bone diseases in human medicine (such as osteoporosis and rickets) is well established, and the interaction between the intestinal microbiota and nutrient absorption has become a focus of research in recent years [3,4]. Although the present study primarily focuses on piglets, it is worth noting that pigs share many physiological similarities with humans in terms of gastrointestinal structure, mineral metabolism, and vitamin D-dependent regulatory pathways [5,6]. Therefore, nutritional strategies aimed at improving mineral absorption and intestinal function in pigs may also provide useful insights into the regulation of bone health in mammals, including humans.

Bone development in piglets is a complex physiological process involving calcium–phosphorus homeostasis, osteoblast proliferation and differentiation, bone matrix deposition, and maintenance of bone mineral density [7,8]. This process is synergistically regulated by multiple factors, including genetics, environment, and nutrition. Among them, nutritional interventions are considered core approaches to improving bone health in piglets due to their feasibility and applicability in production settings [9,10,11]. Vitamin D plays a central role in calcium and phosphorus absorption and skeletal mineralization. In particular, 25-hydroxyvitamin D3 (25-OH-VD_3_), as a major circulating form of vitamin D, contributes to mineral homeostasis by regulating calcium-binding protein (CaBP) expression in intestinal epithelial cells and activating vitamin D receptor (VDR)-mediated signaling pathways in bone-related tissues, thereby promoting calcium and phosphorus deposition and maintaining normal bone mineralization [12]. Phytase, on the other hand, can specifically degrade phytic acid (inositol hexaphosphate) in feed, breaking down the insoluble complexes formed between phytic acid and minerals such as calcium and phosphorus [13]. This significantly improves the utilization of dietary calcium and phosphorus by piglets, reduces bone development retardation caused by insufficient mineral absorption, and simultaneously minimizes environmental pollution from phosphorus emissions [14]. As an important regulator of the intestinal microecology, probiotics not only improve the intestinal flora structure and enhance the intestinal barrier function by secreting organic acids and antimicrobial peptides but also indirectly affect the efficiency of mineral absorption and transport by regulating the intestinal mucosal immune response and promoting the production of short-chain fatty acids (SCFAs) [15]. Furthermore, through the “gut–bone axis” regulatory pathway, probiotics modulate the expression of cytokines involved in bone metabolism (e.g., osteoprotegerin and receptor activator of nuclear factor κB ligand), thereby regulating the dynamic balance between bone formation and bone resorption [12].

Although research on these three factors (25-OH-VD_3_, phytase, and probiotics) in piglet nutrition has gradually deepened, there remains a lack of systematic integration of their synergistic mechanisms of action, dose–effect relationships, and application scenario optimization for the regulation of infant/toddler and piglet bone development. Therefore, this article will focus on the nutritional requirements for piglet bone development, systematically elaborate on the mechanisms by which 25-OH-VD_3_, phytase, and probiotics regulate calcium–phosphorus metabolism and intestinal health in piglets, and analyze the potential advantages of their synergistic application. This study aims to provide theoretical foundations and practical guidance to optimize nutritional strategies for piglet bone development and health, while also offering a cross-species reference for the nutritional regulation of human bone development and health.

## 2. Materials and Methods

### 2.1. Animal Use and Care

All animal experiments and husbandry practices were conducted in strict accordance with institutional ethical guidelines and international regulations governing laboratory animal welfare. The experimental protocols were reviewed and approved by the Institutional Animal Care and Use Committee (IACUC) of Southwest University (approval No. IACUC-20240603-02). Sixty 28-day-old weaned crossbred piglets (Duroc × Landrace × Large White; initial body weight: 7.1 ± 1.30 kg) were sourced from Dekang Agricultural Technology Development Co., Ltd. (Chongqing, China). Piglets were individually housed in stainless-steel metabolic cages measuring 1.8 m × 1.2 m × 0.80 m (length × width × height), with unlimited access to fresh water during the entire experimental period. The rearing room temperature was maintained at approximately 30 °C during the first week, then gradually reduced to 24.0 °C.

### 2.2. Diets and Experimental Design

A total of 60 weaned piglets were randomly allocated to four dietary treatment groups with different supplement combinations, as outlined below: (1) Control group (CON): basal diet supplemented with 50 µg/kg 25-hydroxyvitamin D_3_ (25-OH-VD_3_); (2) 25-OH-VD_3_ + phytase group (HI): CON + 50 µg/kg 25-OH-VD_3_ + 50 mg/kg phytase; (3) 25-OH-VD_3_ + probiotic group (CY): CON + 50 µg/kg 25-OH-VD_3_ + 10 mg/kg probiotic; (4) 25-OH-VD_3_ + phytase + probiotic group (HICY): CON + 50 µg/kg 25-OH-VD_3_ + 50 mg/kg phytase + 10 mg/kg probiotic; experimental additives included phytase (product name: Hiphos), probiotic (product name: Cylactin), and 25-OH-VD_3_ (1.25% HyD, powder), all sourced from DSM (Shanghai, China), with each dietary group comprising 8 replicates of 2 piglets per replicate, a total experimental duration of 31 days (including an initial 3-day adaptation phase to acclimate piglets to the dietary regime and housing environment), a basal diet formulated strictly in line with the nutritional standards recommended by the National Research Council (NRC) [16], and piglets fed twice daily at fixed times (08:00, 12:00 and 18:00), while detailed ingredients and chemical compositions of the experimental diets are provided in Table 1.

### 2.3. Measurements and Sampling

During the experimental phase, piglets’ daily feed consumption was accurately recorded; on trial days 4 and 32, they were weighed post-overnight fasting to ensure consistent conditions, with three key growth performance metrics calculated as follows: Average daily gain (ADG) = total weight gain/(experimental duration × Number of replicates), average daily feed intake (ADFI) = total feed consumed/(experimental duration × number of replicates), and feed conversion ratio (F/G) = ADFI/ADG. From day 25 onward, titanium dioxide (0.3%) was incorporated into the feeds as an indigestible marker, and fecal samples (≈0.2 kg/pig/collection) were collected on days 29–31, mixed with 10% sulfuric acid (10 mL per 100 g feces) to minimize nitrogen and moisture loss, stored at −20 °C until further processing. After collection, replicate samples were pooled, homogenized, freeze-dried, ground into fine powder, and passed through a 1 mm sieve prior to nutrient determination. At the end of the trial, seven replicates per group were randomly selected, with one piglet per replicate chosen for slaughter—after collecting a 5 mL blood sample via anterior vena cava using heparinized vacutainers, and piglets were anesthetized with sodium pentobarbital (50 mg per kg body weight) to ensure humane euthanasia, followed by excision of hind limb metatarsals/phalanges (after dissecting fascia/muscle and rinsing with saline) for skeletal development assessment and carcass dissection to weigh major organs (heart, liver, spleen, lungs, kidneys, pancreas, stomach) for calculating organ indices relative to body weight. For laboratory analyses, 5 cm segments of jejunum and colon were fixed in 4% paraformaldehyde (50 mL centrifuge tubes) for intestinal morphology, a 10 cm colon segment was processed to scrape mucosa for calcium–phosphorus metabolism and immune factor analysis, and a 5 cm colon segment (molecular sample) plus 10 mL colonic contents (gut microbiota profiling) were collected, with all samples flash-frozen in liquid nitrogen and stored at −80 °C for long-term preservation of biological activity.

### 2.4. Analytical Methods

#### 2.4.1. Dietary Compositions

The nutrient composition of the experimental diets was assessed by analyzing dry matter (DM), crude protein (CP), calcium, and total phosphorus, with digestible energy subsequently calculated. For the quantification of DM and CP, the official analytical protocols established by the Association of Official Analytical Chemists (AOAC) were adopted. Specifically, DM content was determined using the AOAC method 930.15 [17], which involves drying samples to constant weight to ensure complete removal of moisture. CP content was analyzed according to the AOAC method 976.05 [17], which is derived from the Kjeldahl nitrogen-determination principle: nitrogen in the sample is converted to ammonia, and the resulting ammonia is quantified to indirectly determine protein content.

The detection of calcium and total phosphorus concentrations adhered to the national standards of the People’s Republic of China. Calcium content was measured in accordance with GB/T 6436-2018 [18], whereas total phosphorus content was analyzed following GB/T 6437-2018 [19]. These national standard methods are designed to ensure the accuracy and uniformity of test results, thereby providing reliable data to support the assessment of key mineral components in the diet.

Dietary digestible energy was calculated using data extracted from the China Feed Database [20]. This database contains comprehensive information on the nutrient composition and energy characteristics of various feed ingredients, enabling accurate computation of digestible energy for specific experimental diets.

#### 2.4.2. Skeletal Performance Measurement

Defatted bone weight. The defatted bone weight was determined using the procedures outlined by Wensley et al. [21]. Frozen bone samples were thawed at room temperature, rinsed with deionized water, and separated into phalanges and metatarsals; after steaming to remove adherent muscles and fascia, the bones were dried to a constant weight, then defatted by immersion in a 2:1 (*v*/*v*) ethanol:benzene mixture for 96 h, and post-defatting, samples were baked at 105 °C to volatilize residual solvents and reweighed to obtain the defatted bone weight.

Bone mineral density (BMD). The bone mineral density was evaluated following the methods described by Keenan et al. [22,23] was measured via Archimedes’ principle at 20 °C: defatted bones were weighed (M), a 250 mL graduated cylinder was filled with distilled water to the 10 mL mark (initial volume V1; cylinder + water mass M1 recorded), bones were added to the cylinder, distilled water was replenished to the minimum measurable scale (final volume V2; cylinder + water + bone mass M2 recorded), and calculations were performed as follows: mass of added water (M3) = M2 − M1 − M, volume of added water (V3) = M3/0.99809 g/cm^3^ (density of distilled water at 20 °C), bone volume (V_bone) = V2 − V1 − V3, and BMD = M/V_bone.

For the analysis of bone ash as well as calcium and phosphorus contents, Bones that had undergone previous measurement of defatted bone weight and bone density were transferred into crucibles and incinerated at 600 °C for 16 h until a white constant weight was achieved [ash content = (ash weight/defatted bone weight) × 100%], calcium content was quantified by EDTA-2Na complexometric titration, and phosphorus content was measured via molybdenum blue colorimetry.

#### 2.4.3. Plasma Biochemical Indicators

A colorimetric assay was used to determine plasma biochemical parameters, including albumin, globulin, glucose, urea, and total cholesterol. All assay kits required for this experiment were sourced from Jiancheng Bioengineering Institute (Nanjing, China). The underlying principle of this colorimetric method is the specific chemical interactions between individual analytes in plasma samples and the reagent components in the kits. These interactions generate colored reaction products, with the color intensity directly proportional to the levels of the measured analytes. Following the reaction, the absorbance of the resulting chromogenic products was recorded, and the plasma biochemical parameters were derived by comparing the measured absorbance with pre-calibrated standard curves.

To quantify immunoglobulins and cytokines, an enzyme-linked immunosorbent assay (ELISA) was selected as the analytical technique. The ELISA kits were acquired from Chenglin Biotechnology Co., Ltd. (Beijing, China). ELISA uses antibodies with high binding affinity for target immunoglobulins and cytokines. After a series of experimental procedures—including sample incubation, washing to remove unbound substances, and substrate-enzyme catalysis—the target analytes bound to the antibodies are detected. The plasma levels of these analytes were then calculated from the standard curves provided with the commercial kits.

#### 2.4.4. Intestinal Mucosa Morphology

Histological examination of fixed jejunal tissue specimens was conducted using hematoxylin-eosin (HE) staining. The experimental protocol was carried out in the following sequence: tissue collection; gradient ethanol dehydration; xylene-mediated clearing; paraffin embedding; section preparation; HE staining; microscopic visualization; and subsequent quantitative analysis of villus height, crypt depth, and goblet cell count.

#### 2.4.5. Quantitative PCR Analysis

Real-time quantitative PCR (qPCR) was utilized to determine the expression profiles of target mRNA in renal tissues and colonic mucosal samples. Primers were designed with Primer Premier 5, and custom-synthesized by Shenggong Biotech Co., Ltd. (Shanghai, China). Detailed sequences of the specific primers are provided in Table S1. Total RNA was extracted from the tissues using a commercial RNA isolation kit (Accurate Biology, Changsha, China) following the manufacturer’s recommended procedures.

Quantitative real-time PCR was performed using a fluorescence-based qPCR kit (Tianjing, China). Each reaction was carried out in a volume of 20 μL containing the following components: 10 μL of 2 × ChamQ SYBR qPCR Master Mix, 0.4 μL of forward primer (10 μM), 0.4 μL of reverse primer (10 μM), 0.4 μL of 50 × ROX Reference Dye 1, 2 μL of cDNA template, and 6.8 μL of nuclease-free water. The thermal cycling program was set as follows: initial enzyme activation at 95 °C for 2 min, followed by 40 amplification cycles consisting of denaturation at 95 °C for 15 s, annealing and extension at 60 °C for 60 s, and a final fluorescence collection step at 60 °C for 10 s.

Relative quantification of target gene expression was calculated using the 2^−△△Ct^ method. The formula for calculation is defined as follows: Relative expression level of target gene = 2^−△△Ct^; where △△Ct = (Ct value of target gene − Ct value of internal reference gene) in the experimental group − (Ct value of target gene − Ct value of internal reference gene) in the control group.

#### 2.4.6. Western Blotting

Renal tissue-derived total protein was analyzed by Western blotting according to standardized protocols, including SDS-PAGE, transfer to a PVDF membrane, blocking, incubation with the primary antibody, incubation with the secondary antibody, and enhanced chemiluminescence (ECL) detection. For SDS-PAGE, 20 μg of normalized protein per sample was resolved alongside a pre-stained protein ladder (Bio-Rad, Hercules, CA, USA) as a molecular weight reference. Separated proteins were electrotransferred onto a PVDF membrane, then blocked with 5% (*w*/*v*) BSA in TBST at room temperature for 1 h to reduce non-specific binding. Primary antibodies were applied to the membranes and incubated overnight at 4 °C, including anti-β-actin (8115-1-RR; Proteintech, Rosemont, IL, USA; diluted 1:5000), anti-cytochrome P450 27B1 (CYP27B1) (PA5-79128; Thermo Fisher Scientific, Waltham, MA, USA; diluted 1:2000), and anti-CaSR (19125-1-AP; Proteintech; diluted 1:1000). After three 10 min TBST washes to remove unbound primary antibodies, membranes were incubated with a recombinant rabbit secondary antibody (RGAR001; Proteintech) at 1:5000 dilution for 1 h at ambient temperature. Protein bands were visualized using an ECL detection system (ChemiDoc™ Imaging System, Bio-Rad) and quantified using ImageJ software (version 1.53g, NIH, Bethesda, MD, USA). β-actin served as the internal loading control for normalizing protein expression across samples.

#### 2.4.7. Intestinal Microbiota

The microbial lineage analysis was entrusted to Shanghai Meiji Biotechnology Co., Ltd. (Shanghai, China). Microbial diversity was determined on the Illumina NovaSeq platform, which uses paired-end (double-end) sequencing. This method involves constructing small-fragment libraries, a crucial step that fragments genomic DNA from microbial samples to the appropriate size and attaches specific adapters to both ends. These prepared libraries are then ready for high-throughput sequencing, enabling the simultaneous sequencing of millions of DNA fragments from the microbial samples.

Following sequencing, a series of bioinformatics analyses are carried out to characterize the microbial species composition. First, the obtained reads are carefully spliced together, and low-quality reads are filtered out to ensure data accuracy. Subsequently, the processed sequences undergo clustering or denoising operations. Clustering similar sequences into operational taxonomic units (OTUs) and denoising directly remove noise sequences; both aim to accurately represent the true microbial diversity. After that, species annotation is performed by comparing the processed sequences against well-established reference databases that assign taxonomic identities. Meanwhile, abundance analysis quantifies the relative proportion of each microbial species in the samples, providing insights into the microbial community structure.

To further explore the differences between samples, multiple analytical approaches are employed. Alpha-diversity metrics, such as the Shannon and Simpson indices, are used to evaluate diversity within a single sample and reflect the richness and evenness of the microbial community. Beta diversity analysis, including methods such as Principal Coordinate Analysis (PCoA) and Non-metric Multidimensional Scaling, compares dissimilarity among multiple samples, revealing compositional differences among microbial communities. Additionally, significant species differences are identified using statistical tests to pinpoint the microbial species contributing to the observed variation. Correlation analysis uncovers relationships among microbial species or between microbial species and environmental factors, whereas functional prediction, often based on metagenomic data, infers the potential functions and metabolic pathways of the microbial community.

The overall microbial detection process encompasses several key steps. It begins with the extraction of genomic DNA from the samples, a critical procedure that isolates microbial DNA while removing contaminants. Subsequently, PCR amplification is performed to replicate the target DNA regions, followed by purification of the product to remove amplification-derived contaminants. The purified PCR products are then accurately quantified, and homogenization is performed to ensure consistent input amounts for the next step. Next, sequencing libraries are constructed, as previously described, to prepare the samples for sequencing. Bipartite sequencing on the Illumina NovaSeq platform generates the raw sequencing data. Finally, analyses of sample diversity and differences, as mentioned above, are conducted to provide a comprehensive understanding of microbial community characteristics and variation among samples.

### 2.5. Statistical Analysis

Experimental data were processed using Excel 2023 for organization and computation. Statistical analyses were performed with SPSS 20.0. A mixed-effects model was applied as follows: *Y_ij_* = *µ* + *T_i_* + *S_j_* + *ɛ_ij_*, where *Y_ij_* denotes the measured response variable, *µ* represents the overall mean, *T_i_* is the fixed effect of dietary treatment, *S_j_* is the random effect of pig, and *ɛ_ij_* indicates the residual error. Duncan’s method was used for multiple comparisons, and results were expressed as means and standard errors (SEM). Statistical significance was set at *p* < 0.05.

## 3. Results

### 3.1. Growth Performance and Nutrient Apparent Digestibility

Table 2 summarizes the growth performance and apparent nutrient digestibility of weaned piglets. No statistically significant differences (*p* > 0.05) were detected across all experimental groups for key growth indicators, including body weight gain, ADG, ADFI, and F/G. The dry matter apparent digestibility of piglets in the CON group was higher than that of piglets in the HICY group (*p* < 0.05). For CP apparent digestibility, a significant difference was observed among the four groups (*p* = 0.002), with the CON group showing notably higher values than the HI and HICY groups (*p* > 0.05). The CON group exhibited significantly lower calcium digestibility than the HI, CY, and HICY groups (*p* > 0.05).

### 3.2. Organ Index

As presented in Table 3, no significant differences were observed in organ indices of the heart, spleen, pancreas, liver, lung, or stomach among the four experimental groups at trial completion (*p* > 0.05). Notably, the kidney index in the HICY group was significantly greater than that in the CON, HI, and CY groups (*p* < 0.005).

### 3.3. Serum Biochemical Indices

As presented in Table 4, there were no significant disparities in the serum concentrations of albumin, globulin, glucose, blood urea nitrogen, serum calcium, serum phosphorus, tartrate-resistant acid phosphatase, bone alkaline phosphatase, or parathyroid hormone across the four piglet groups (*p* > 0.05). The serum 1,25-(OH)_2_-D_3_ concentration of piglets in the CON group was significantly lower than that in the HI and CY (*p* < 0.001). Compared to the CON diet, the HI and CY diets significantly enhanced the serum 25-OH-VD_3_ activity in weaned piglets (*p* < 0.05).

### 3.4. Serum Cytokine Indices

As presented in Table 5, the CY and HICY groups presented a markedly higher serum TGF-β concentration than the CON and HI groups (*p* < 0.05). Piglets in the CON and HI groups displayed a significantly elevated level of serum IL-1β compared to the piglets in the HI, CY, and HICY groups (*p* < 0.05). The serum concentrations of IL-8, IL-10, TNF-α, IgA, IgG, and IgM in piglets did not differ significantly among the four dietary treatments (*p* > 0.05).

### 3.5. Bone Performance

As presented in Table 6, piglets in the HI group exhibited the highest metatarsal BMD, which was significantly greater than that observed in the CON, CY, and HICY groups (*p* < 0.05). Calcium content of the metatarsal in the HI and HICY groups was also significantly elevated compared with that in the CON group (*p* < 0.05). However, no statistically significant differences in the skimmed bone weight and phosphorus and crude ash contents of the metatarsal were observed among the four groups (*p* > 0.05).

For the toe bones, BMD was highest in the HI group, showing a significant increase compared with the CON, CY, and HICY groups (*p* < 0.05). The HI, CY, and HICY groups had the same calcium content, which was significantly higher than that of the CON group (*p* < 0.05). No significant difference was detected in toe bone skimmed weight and phosphorus and crude ash contents of toe bone among groups (*p* > 0.05).

### 3.6. Serum Oxidative Stress Markers

As presented in Table 7, the serum T-AOC level was notably lower in the CON group compared with the HI, CY, and HICY groups (*p* < 0.05), and the CY group exhibited the highest serum T-AOC among the four groups (*p* < 0.05). No significant differences were found among the four groups for serum superoxide dismutase (SOD), malondialdehyde (MDA), or glutathione peroxidase (GSH-PX) among the four groups (*p* > 0.05).

### 3.7. Intestinal Mucosal Morphology

**Figure 1:**
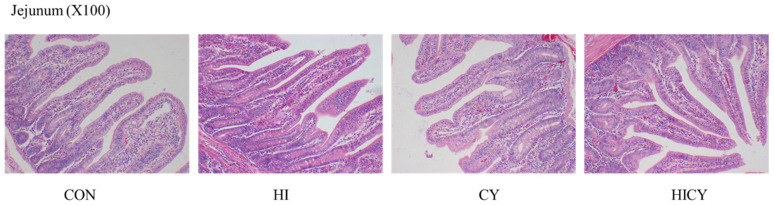
Effects of dietary 25-OH-VD3 combined with phytase and probiotic on the jejunum mucosal morphology of weaned piglets. Data are presented as means (*n* = 6).

As shown in Table 8 and Figure 1, no significant differences in jejunal villus height, crypt depth, or V/C were observed among groups (*p* > 0.05).

### 3.8. Calcium and Phosphorus Metabolism-Related mRNA Expression

**Figure 2:**
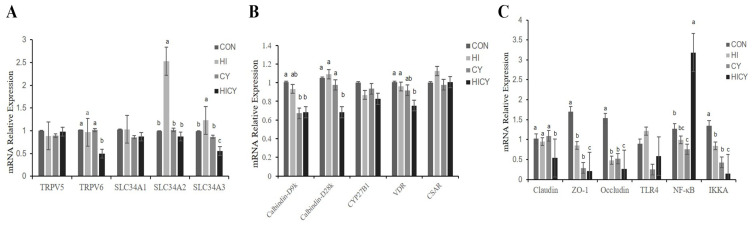
Effects of dietary 25-OH-VD_3_ combined with phytase and probiotic on the relative mRNA expression in the jejunal mucosa for calcium and phosphorus metabolism in weaned piglets. (**A**) Calcium and phosphate transporter gene expression in jejunal mucosa; (**B**) Vitamin D metabolism and calcium-binding gene expression in jejunal mucosa; (**C**) Intestinal barrier function and inflammation-related gene expression in jejunal mucosa. Data are expressed as means (*n* = 6). ^a,b,c^ Values within the same row with different superscript letters indicate significant differences (*p* < 0.05).

As presented in Figure 2, the relative mRNA expression of transient receptor potential cation channel subfamily V member 6 (*TRPV6*) in the jejunal mucosa of piglets in the HICY group was lower than that of piglets in the CON, HI, and CY groups (*p* < 0.05). The relative mRNA expression of solute carrier family 34, member 2 (*SLC34A2*) and *SLC34A3* in the jejunal mucosa of piglets in the HI group was significantly higher than in the CON, CY, and HICY groups (*p* < 0.05). The relative mRNA expression of calcium-binding protein D9k (*Calbindin-D9k*) in the jejunal mucosa of piglets in the CY and HICY groups was significantly lower than in the CON group (*p* < 0.05). Compared with pigs fed the CON, HI, and CY diets, those fed the HICY diet showed reduced relative mRNA expression of calcium-binding protein D28k (*Calbindin-D28k*) (*p* < 0.05). The relative mRNA expression of *VDR* was significantly decreased in the HICY group relative to the CON and HI groups (*p* < 0.05). The relative mRNA expression of claudin, zonula occludens-1 (*ZO-1*), occludin, and IκB kinase α (*IKKA*) in the jejunal mucosa was significantly lower in the HICY group compared with the CON, HI, and CY groups (*p* < 0.05). The relative mRNA expression of *IKKA* also reduced in the HI and CY groups relative to the CON group (*p* < 0.05). Conversely, the relative mRNA expression of nuclear factor-κB (*NF-κB*) was elevated in the HICY group compared with the CON, HI, and CY groups (*p* < 0.05), whereas the relative mRNA expression of *NF-κB* in the CY group was significantly lower than that of the CON group (*p* < 0.05).

**Figure 3:**
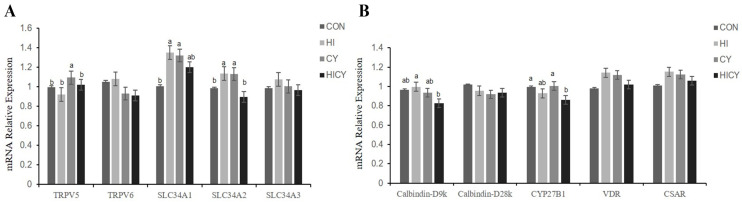
Effects of dietary 25-OH-VD_3_ combined with phytase and probiotic on the relative mRNA expression for calcium and phosphorus metabolism in the kidney of piglets. (**A**) Calcium and phosphate transporter gene expression in kidney; (**B**) Vitamin D metabolism and calcium-binding gene expression in kidney. Data are presented as mean and SEM (*n* = 6). ^a,b^ Values within a row with different superscripts differ significantly (*p* < 0.05).

As shown in Figure 3, the relative mRNA expression of *TRPV5* in the kidney was significantly upregulated in the CY group compared with the CON, HI, and HICY groups (*p* < 0.05). In addition, the relative mRNA expression of *SLC34A1* in the kidney of piglets was markedly higher in the HI and CY groups than in the CON group (*p* < 0.05). There was an increase in the relative mRNA expression of *SLC34A2* in the kidney in the HI and CY groups compared with those of pigs in the CON and HICY groups (*p* < 0.05). The relative mRNA expression of *Calbindin-D9k* in the kidney of piglets in the HICY group was significantly lower than in the HI group (*p* < 0.05). The relative mRNA expression of *CYP27B1* in the kidney of piglets in the HICY group was significantly lower than in the CON and CY groups (*p* < 0.05).

### 3.9. Calcium and Phosphorus Metabolism-Related Protein Expression

**Figure 4:**
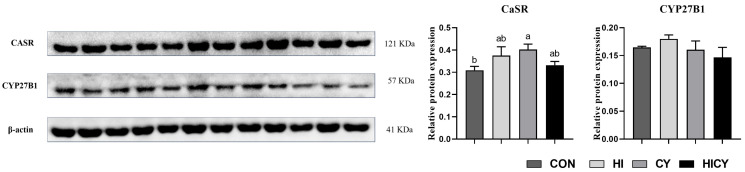
Effects of dietary 25-OH-VD_3_ combined with phytase and probiotic on renal protein expression related to calcium and phosphorus metabolism in weaned piglets. Data are expressed as means (*n* = 4). ^a,b^ Values within the same row with different superscript letters indicate significant differences (*p* < 0.05).

As shown in Figure 4, the protein expression of CaSR in the kidney of piglets in the CY group was significantly elevated compared to that in the CON group (*p* < 0.05). There were no significant differences in the protein expression of CYP27B1 in the kidney of piglets among the four groups (*p* > 0.05).

### 3.10. Colon Microbiota

**Figure 5:**
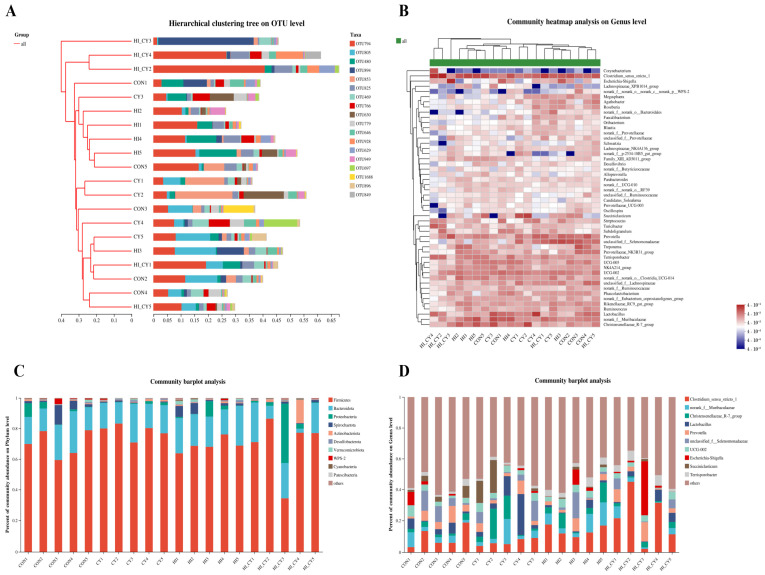
Effects of dietary 25-OH-VD_3_ combined with phytase and probiotic on the colonic microbiota of weaned piglets (*n* = 5). (**A**) Hierarchical clustering tree at the OTU level. (**B**) Community heatmap analysis at the genus level. (**C**) Community barplot analysis at the phylum level. (**D**) Community barplot analysis at the genus level. Abbreviations: OTUs, operational taxonomic units; PCoA, principal coordinate analysis.

As shown in Figure 5A, the samples within the same group exhibited tight clustering at the OTU level, reflecting strong intra-group consistency. The HI and CY groups showed some cross-clustering, whereas the HICY (combined treatment) group was more distantly related to the CON group. Community heatmap analysis at the genus level is shown in Figure 5B. The HICY group had the highest abundance of beneficial bacteria, including *Macrocystis* and *Agathobacter* (*p* < 0.05). The abundances of *Prevotella* (linked to carbohydrate metabolism) and *Blautia* in the CY group were similar to those in the CON group (*p* > 0.05). Community barplot analysis at the phylum level is shown in Figure 5C. Firmicutes and Bacteroidetes together accounted for over 80% of the total bacterial population. The HICY group exhibited a relatively high proportion of Proteobacteria (15–20%), which are associated with harmful bacteria, and a lower proportion of Actinobacteria (2–3%), which are associated with beneficial bacteria. The CY group displayed a Bacteroidetes proportion (35–40%) similar to that of the CON group. Community barplot analysis at the genus level is shown in Figure 5D; the CON group had a high abundance of beneficial bacteria (*Lactobacillus* and *Christensenellaceae*) (*p* < 0.05), and a lower abundance of potentially harmful bacteria (*Clostridium* and *Escherichia coli/Shigella*) (*p* < 0.05); the HICY group shows the opposite, with reduced flora diversity (*p* < 0.05).

## 4. Discussion

**Figure 6:**
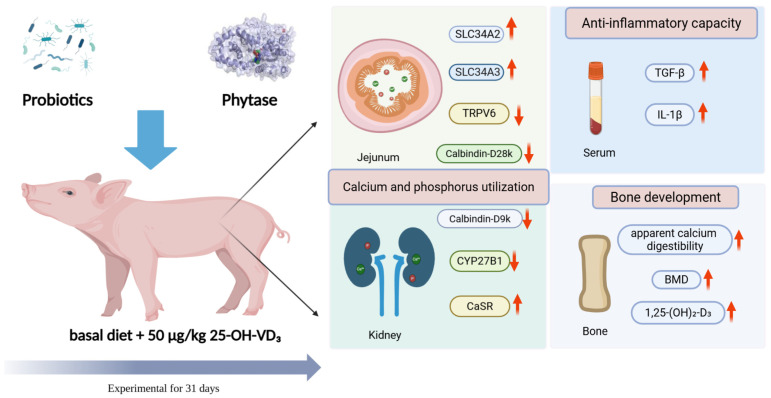
Effects of dietary 25-OH-VD_3_ combined with phytase and probiotics on calcium–phosphorus metabolism and bone development in weaned piglets. Abbreviations: SLC34A2/3, solute carrier family 34 (type II sodium/phosphate transporter), member 2/3; TRPV6, transient receptor potential cation channel subfamily V member 6; Calbindin-D9k, calcium-binding protein D9k; Calbindin-D28k, calcium-binding protein D28k; CYP27B1, cytochrome P450 27B1; CaSR, calcium sensing receptor; TGF-β, Transforming Growth Factor-β; IL-1β, Interleukin-1β; BMD, bone mineral density.

This study systematically evaluated the effects of 25-hydroxyvitamin D_3_ (25-OH-VD_3_) combined with phytase and probiotics on calcium–phosphorus metabolism, bone development, immune function, oxidative stress responses, and intestinal microbiota in weaned piglets (Figure 6). The results revealed that dietary supplementation with these additives differentially regulated nutrient utilization, molecular signaling, and microbial homeostasis, with distinct advantages of single probiotic addition (CY group) over combined treatments (HICY group) in practical applications.

### 4.1. Effects on Calcium–Phosphorus Metabolism and Bone Development

Bone development in weaned piglets is tightly coupled with calcium–phosphorus metabolism, and nutritional interventions targeting this process are critical for addressing weaning stress-induced mineral imbalances [6]. In the present study, the HI (25-OH-VD_3_ + phytase) and HICY groups exhibited significantly higher Ca apparent digestibility than the CON group, consistent with the core function of phytase in degrading phytic acid—an anti-nutrient that forms insoluble complexes with calcium–phosphorus [13]. Phytase-mediated release of bound minerals directly improves their bioavailability, which was further reflected in the HI group’s superior metatarsal and toe BMD and metatarsal calcium content. This aligns with Lautrou et al. [14], who reported that phytase supplementation enhances bone mineralization by increasing P utilization in growing pigs.

Notably, the CY group (25-OH-VD_3_ + probiotic) also showed elevated calcium digestibility and toe bone calcium content, which can be attributed to the “gut–bone axis” regulatory pathway [15]. Probiotics modulate intestinal mucosal immunity and barrier function, promoting the absorption of fat-soluble vitamins (including 25-OH-VD_3_) by reducing intestinal inflammation and enhancing epithelial integrity [12]. The significantly higher serum 1,25-(OH)_2_-D_3_ and 25-OH-VD_3_ levels in the CY group further confirmed this mechanism: 1,25-(OH)_2_-D_3_, the active form of vitamin D, upregulates intestinal calcium transporters (e.g., TRPV5) and CaBPs to facilitate calcium absorption [10].

In contrast, the HICY group (combined three additives) did not show additive benefits in bone BMD compared to the HI group, and even exhibited lower metatarsal BMD than the HI group. This may be due to antagonism between phytase and probiotics: phytase-induced changes in intestinal pH or nutrient composition may affect probiotic viability [24], whereas excessive phosphorus release from phytase could disrupt the calcium-to-phosphorus ratio, thereby indirectly impairing bone mineralization [8]. Additionally, the HICY group’s significantly higher kidney index suggests altered renal function, which may be associated with the need to excrete excess minerals or adapt to molecular regulatory changes.

### 4.2. Regulation of Molecular Signaling in Calcium–Phosphorus Metabolism

The expression of genes and proteins involved in calcium–phosphorus transport and vitamin D activation provides insights into the underlying regulatory networks. In the kidney, the HICY group showed significantly lower mRNA expression of *Calbindin-D9k* and *CYP27B1*. Calbindin-D9k is a key Ca-binding protein that facilitates calcium reabsorption in the distal tubule [25], and its downregulation may reflect a negative feedback response to elevated serum calcium levels or altered vitamin D signaling. CYP27B1 is the rate-limiting enzyme for converting 25-OH-VD_3_ to 1,25-(OH)_2_-D_3_ [26]; the reduced CYP27B1 mRNA and protein expression in the HICY group is consistent with the moderate serum 1,25-(OH)_2_-D_3_ levels (lower than the CY group), indicating that combined treatments may dampen vitamin D activation to avoid excessive calcium–phosphorus deposition.

The higher relative CaSR protein expression in the HI, CY, and HICY groups compared with the CON group underscores CaSR’s role in maintaining calcium homeostasis. CaSR detects extracellular calcium concentrations and regulates renal calcium excretion and bone resorption [7]; its upregulation suggests increased sensitivity to calcium levels, which is crucial for preventing calcium overload in response to improved calcium digestibility.

In the colon, the HI group’s higher *SLC34A2* mRNA expression (a key phosphorus transporter) directly supports the observed increase in phosphorus utilization, as SLC34A2 mediates intestinal phosphorus absorption [13]. However, the HICY group showed lower expression of *Calbindin-D28k* and *VDR* in the colon, which may explain its lack of additive benefits—VDR is essential for vitamin D-mediated regulation of calcium and phosphorus transporters [10], and reduced VDR expression could impair intestinal epithelial cell responsiveness to 25-OH-VD_3_.

### 4.3. Immune Function and Oxidative Stress Responses

Weaned piglets are highly susceptible to inflammation and oxidative stress due to weaning-induced intestinal dysfunction [5]. The CY group’s significantly higher serum TGF-β levels and lower IL-1β levels indicate that probiotics exert anti-inflammatory effects. TGF-β is a key anti-inflammatory cytokine that inhibits pro-inflammatory pathways (e.g., NF-κB) and promotes intestinal barrier repair [27], while reduced IL-1β (a pro-inflammatory cytokine) suggests mitigated intestinal inflammation. This aligns with de Sire et al. [15], who reported that probiotics regulate the gut–bone axis by modulating cytokine profiles, thereby reducing bone resorption.

The CY group also exhibited the highest serum total antioxidant capacity (T-AOC), suggesting enhanced resistance to oxidative stress. Probiotics secrete antioxidants (e.g., glutathione) and regulate host antioxidant enzyme activity [28], which helps protect intestinal epithelial cells from oxidative damage and maintain nutrient absorption capacity. In contrast, the HICY group did not exhibit superior immune or antioxidant performance, possibly because phytase-induced changes in intestinal microbiota composition counteract the beneficial effects of probiotics.

### 4.4. Intestinal Morphological Development

Jejunal villus height and V/C ratio are key indicators of intestinal mucosal integrity and nutrient absorption capacity—longer villi increase the absorption surface area, while a higher V/C ratio reflects mature intestinal epithelial function and reduced mucosal damage [5].

The numerical increase in villus height and V/C ratio in the CY group aligns with the probiotic’s role in regulating intestinal health. Probiotics secrete SCFAs and antimicrobial peptides, which enhance intestinal barrier function by promoting tight junction protein expression and reducing inflammation [15]. This may explain why the CY group maintained more favorable intestinal morphology, consistent with its superior serum anti-inflammatory cytokine (TGF-β) levels and a balanced gut microbiota (e.g., higher Lactobacillus abundance) as observed in previous studies.

In contrast, the HI group showed a trend toward reduced villus height, which may be related to phytase-induced changes in the intestinal environment. Phytase degrades phytic acid to release phosphorus, but this process can alter intestinal pH [24] or interfere with mineral binding to mucosal proteins, thereby indirectly affecting epithelial cell proliferation. The HICY group’s intermediate villus height suggests a potential antagonism between phytase and probiotics: phytase-mediated pH shifts may reduce probiotic viability, while probiotics’ metabolic products could inhibit phytase activity, leading to a blunted effect on intestinal morphology compared to the CY group.

The lack of significant differences in crypt depth among groups indicates that dietary treatments did not disrupt intestinal epithelial cell renewal. However, the near-significant trend in villus height (*p* = 0.064) suggests that longer experimental periods or adjusted additive doses may reveal more pronounced effects. Overall, probiotic supplementation shows promise for improving intestinal mucosal morphology in weaned piglets, while the combination of phytase and probiotics does not provide additive benefits, possibly due to their incompatible effects on the intestinal microenvironment.

### 4.5. Modulation of Intestinal Microbiota

The intestinal microbiota plays a pivotal role in nutrient metabolism and host health through the gut–bone axis [29]. The present study revealed that dietary treatments significantly altered the structure of the colonic microbiota in weaned piglets. The CY group maintained a microbiota profile most similar to that of the CON group, with higher abundances of beneficial genera (e.g., Lactobacillus, Prevotella) and a comparable proportion of Bacteroidota. Lactobacillus produces SCFAs that enhance intestinal barrier function and calcium absorption [30], while Prevotella is involved in carbohydrate metabolism to provide energy for intestinal epithelial cells [31]. This explains why the CY group showed optimal performance in Ca-P metabolism and immune function.

In contrast, the HICY group exhibited the most distinct microbiota structure, with higher proportions of harmful Proteobacteria (15–20%) and lower proportions of beneficial Actinobacteria (2–3%). Proteobacteria overgrowth is associated with intestinal inflammation and barrier dysfunction [32], which may contribute to the HICY group’s altered gene expression (e.g., higher colon NF-κB) and reduced bone BMD. The HI group showed moderate changes in microbiota, with limited effects on health-related taxa, suggesting that phytase primarily affects microbial metabolic pathways rather than community structure [9]. These results suggest that combining phytase with probiotics may disrupt microbial homeostasis, underscoring the importance of evaluating additive compatibility in feed formulation [24].

### 4.6. Growth Performance

Notably, no significant differences in growth performance (ADG, ADFI, F/G) were observed among groups, which may be attributed to the short experimental period (31 days) and the priority of nutrient allocation to bone development and metabolic adaptation during the weaning stage [5]. Bone development is a long-term process, and the benefits of nutritional interventions may become more evident in later growth stages [11]. Additionally, the basal diet was formulated to meet the NRC requirements [16]; therefore, the marginal improvements in nutrient digestibility may not have been sufficient to drive significant differences in growth.

From a practical perspective, the CY group (single probiotic addition) showed the most balanced performance: improved Ca-P metabolism, enhanced immune function, maintained intestinal microbiota homeostasis, and no adverse effects on organ indices. In contrast, the HICY group’s disrupted microbiota and altered renal function suggest that combined supplementation may not be optimal for weaned piglets. The HI group’s higher bone BMD indicates that phytase is effective in improving bone mineralization; however, its lack of immune and microbiota benefits limits its application in comprehensive health management. These findings provide a theoretical basis for optimizing weaned piglet feed formulations—single-probiotic supplementation is recommended to promote calcium–phosphorus metabolism and intestinal health, while phytase may be preferred to target bone development.

## 5. Conclusions

In this study, single probiotic supplementation improved calcium and phosphorus utilization and bone development and enhanced antioxidant capacity of piglets; single phytase supplementation effectively improved calcium and phosphorus utilization and bone mineralization and enhanced antioxidant and anti-inflammatory capacity of piglets; combined supplementation improved calcium and phosphorus utilization and bone development and enhanced antioxidant and anti-inflammatory capacity of piglets. Overall, 25-OH-VD_3_ combined with phytase and probiotics differentially affects calcium–phosphorus metabolism, bone development, immune function, and intestinal microbiota in weaned piglets. It should be noted that these findings, derived from weaned piglets, may have implications for human bone health. The observed improvement in BMD, potentially mediated by enhanced calcium digestibility and vitamin D activation, suggests that targeted nutritional strategies combining vitamin D metabolites with phytase or probiotics warrant further investigation to support bone accrual in at-risk populations. However, the translational relevance of these results requires validation in human studies.

## Figures and Tables

**Table 1:** The ingredients and composition of diets (dry matter basis, %).

Items	Treatments			
	CON	HI	CY	HICY
Ingredients				
Corn	61.49	61.49	61.49	61.49
Soybean meal	12	12	12	12
Extruded full-fat soybean	6.08	6.08	6.08	6.08
Whey powder	5	5	5	5
Fatty powder	1.59	1.59	1.59	1.59
Fish meal	5	5	5	5
Glucose	5	5	5	5
L-Lys HCL (78%)	0.93	0.93	0.93	0.93
CaCO_3_	0.64	0.64	0.64	0.64
Extruded full-fat soybean	6.08	6.08	6.08	6.08
CaHPO_4_·2H_2_O	0.57	0.57	0.57	0.57
DL-Met (99%)	0.21	0.21	0.21	0.21
L-Thr (98.5%)	0.2	0.2	0.2	0.2
NaCl	0.3	0.3	0.3	0.3
Premix ^1^	1	1	1	1
Chemical composition				
Metabolic energy ^2^	13.9	13.9	13.9	13.9
Crude protein ^3^	18.50	18.50	18.50	18.50
Calcium ^3^	0.63	0.63	0.63	0.63
Phosphorus ^3^	0.35	0.35	0.35	0.35

^1^ Premix supplied the following nutrients per kilogram of diet: Cu (from copper sulfate), 100 mg; Fe (from ferrous sulfate), 100 mg; Zn (from zinc oxide), 120 mg; Mn (from manganese sulfate), 20 mg; I (from calcium iodate), 0.3 mg; and Se (from sodium selenite), 0.3 mg; vitamin A, 3, 800 IU; 25-OH-D3, 50 µg/kg; vitamin E, 10 IU; vitamin K, 1 mg; choline, 200 mg; pantothenic, 5 mg; vitamin B2, 2 mg; folic acid, 0.8 mg; vitamin B1, 1 mg; vitamin B6, 1 mg; biotin, 0.08 mg; vitamin B12, 0.01 mg. ^2^ Calculated values. ^3^ Analyzed values. Abbreviations: CON, basal diet supplemented with 50 µg/kg 25-OH-VD_3_; HI, CON + 50 mg/kg phytase; CY, CON + 10 mg/kg probiotic; HICY, CON + 50 mg/kg phytase + 10 mg/kg probiotic. These abbreviations are consistent with those used in the tables and figures below.

**Table 2:** Effects of dietary 25-OH-VD_3_ combined with phytase and probiotics on the growth performance and nutrient apparent digestibility of weaned piglets (*n* = 10).

Items	Treatments				SEM	*p*-Value
	CON	HI	CY	HICY		
Growth performance						
Initial weight (kg)	7.36	7.11	7.11	7.13	0.24	0.983
Day 14 weight (kg)	9.45	9.37	9.40	9.44	0.41	0.986
Day 28 weight (kg)	14.5	14.6	14.7	14.8	0.53	0.968
Day 1–14						
ADG (g)	149	161	164	151	7.51	0.959
ADFI (g)	264	268	270	270	4.40	0.512
Feed: Gain	1.77	1.66	1.65	1.64	0.67	0.451
Day 15–28						
ADG (g)	361	374	379	383	8.19	0.147
ADFI (g)	694	698	705	708	12.02	0.625
Feed: Gain	1.92	1.87	1.86	1.85	0.13	0.221
Day 1–28						
ADG (g)	255	268	271	274	5.71	0.197
ADFI (g)	479	483	488	491	7.15	0.205
Feed: Gain	1.88	1.80	1.80	1.79	0.06	0.178
Nutrient apparent digestibility						
Dry matter, %	94.9 ^a^	93.3 ^ab^	93.2 ^ab^	92.6 ^b^	0.37	0.031
Crude protein, %	78.5 ^a^	75.6 ^b^	76.9 ^ab^	74.6 ^b^	1.05	0.002
Calcium, %	57.3 ^c^	60.2 ^b^	60.4 ^b^	65.4 ^a^	1.64	0.005
Phosphorus, %	68.0	69.0	65.9	68.8	0.83	0.364

Data are expressed as means. ^a,b,c^ Values within the same row with different superscript letters indicate significant differences (*p* < 0.05).

**Table 3:** Effects of dietary 25-OH-VD_3_ combined with phytase and probiotics on organ index of weaned piglets (*n* = 7).

Items	Treatments				SEM	*p*-Value
	CON	HI	CY	HICY		
Organ weight, g						
Heart	52.4	56.2	52.5	59.0	1.98	0.616
Liver	288	305	310	306	11.9	0.925
Spleen	22.0	20.67	21.3	23.3	0.97	0.825
Lung	133	132	125	134	4.63	0.902
Kidney	46.4	49.5	44.3	55.8	1.72	0.091
Pancreas	18.0	20.8	20.3	21.7	1.10	0.682
Stomach	93.1	97.8	104	90.3	3.73	0.636
Organ index, g/kg						
Heart	4.49 ^ab^	4.18 ^b^	4.01 ^b^	5.14 ^a^	0.14	0.023
Liver	24.3	22.8	23.5	26.5	0.64	0.200
Spleen	1.92	1.53	1.65	2.08	0.10	0.243
Lung	11.5	9.83	9.61	11.76	0.39	0.111
Kidney	4.04 ^b^	3.69 ^b^	3.39 ^b^	4.89 ^a^	0.17	0.005
Pancreas	1.54	1.52	1.55	1.93	0.09	0.321
Stomach	7.84	7.29	7.86	7.85	0.16	0.526

Data are expressed as means. ^a,b^ Values within the same row with different superscripts letters indicate significant differences (*p* < 0.05).

**Table 4:** Effects of dietary 25-OH-VD_3_ combined with phytase and probiotics on the serum biochemical indices of weaned piglets (*n* = 7).

Items	Treatments				SEM	*p*-Value
	CON	HI	CY	HICY		
Albumin, g/L	25.2	24.9	27.8	26.2	1.484	0.394
Globulin, μg/mL	13.44	12.60	13.61	13.70	0.689	0.738
Glucose, mmol/L	7.52	6.81	5.93	6.01	0.579	0.426
Blood urea nitrogen, mmol/L	3.30	3.29	1.96	3.20	0.510	0.069
Total cholesterol, g/L	1.92	1.82	1.79	1.54	0.125	0.480
Calcium, mmol/L	2.39	2.84	2.59	2.36	0.167	0.491
Phosphorus, mmol/L	2.26	2.30	2.30	2.24	0.046	0.696
1,25-(OH)_2_-D_3_, ng/L	315 ^b^	350 ^a^	355 ^a^	334 ^b^	6.432	0.001
25-OH-VD_3_, μg/L	13.2 ^b^	14.8 ^a^	15.6 ^a^	14.3 ^ab^	0.767	0.008
Anti-tartrate acid phosphatase, ug/L	6.83	6.13	9.25	6.00	2.07	0.842
Bone Alkaline Phosphatase, ug/L	2.17	1.58	3.46	1.62	1.029	0.231
Parathyroid hormone, ng/L	13.08	13.08	13.04	13.12	0.135	0.782

Data are expressed as means. ^a,b^ Values within the same row with different superscript letters indicate significant differences (*p* < 0.05).

**Table 5:** Effects of dietary 25-OH-VD_3_ combined with phytase and probiotics on the serum cytokine indices of weaned piglets (*n* = 6).

Items	Treatments				SEM	*p*-Value
	CON	HI	CY	HICY		
IL-1β, ng/L	444 ^a^	421 ^a^	383 ^b^	379 ^b^	14.2	0.005
IL-8, ng/L	34.5	31.4	30.7	30.4	0.86	0.069
IL-10, ng/L	24.5	23.2	26.6	26.0	1.07	0.355
TGF-β, ng/L	146 ^b^	153 ^b^	203 ^a^	195 ^a^	11.0	0.001
TNF-α, ng/L	282	266	275	255	10.8	0.201
IgA, μg/L	56.7	55.0	65.0	61.8	2.42	0.103
IgG, μg/L	354	385	357	386	19.7	0.076
IgM, μg/L	76.6	74.0	86.5	81.0	2.32	0.437

Data are expressed as means. ^a,b^ Values within the same row with different superscript letters indicate significant differences (*p* < 0.05). Abbreviations: IL-1β, Interleukin-1β; IL-8, Interleukin-8; IL-10, Interleukin-10; TGF-β, Transforming Growth Factor-β; TNF-α, Tumor Necrosis Factor-α; IgA, Immunoglobulin A; IgG, Immunoglobulin G; IgM, Immunoglobulin M.

**Table 6:** Effects of dietary 25-OH-VD_3_ combined with phytase and probiotic on the bone performance of weaned piglets (*n* = 6).

Items	Treatments				SEM	*p*-Value
	CON	HI	CY	HICY		
Metatarsal						
BMD, g/mL	0.98 ^b^	1.48 ^a^	1.08 ^b^	1.16 ^b^	0.043	<0.001
Skimmed bone weight, g	8.18	7.04	7.04	7.34	0.211	0.183
Calcium, %	0.16 ^b^	0.18 ^a^	0.17 ^ab^	0.18 ^a^	0.003	0.001
Phosphorus, %	0.07	0.08	0.07	0.08	0.002	0.548
Crude ash, %	0.42	0.42	0.42	0.41	0.002	0.673
Toe bone						
BMD, g/mL	0.32 ^c^	0.60 ^a^	0.40 ^b^	0.49 ^b^	0.03	0.001
Skimmed bone weight, g	3.62	3.95	3.95	4.00	0.095	0.48
Calcium, %	0.17 ^b^	0.19 ^a^	0.19 ^a^	0.19 ^a^	0.002	<0.001
Phosphorus, %	0.07	0.07	0.07	0.07	0.002	0.948
Crude ash, %	0.43	0.43	0.42	0.42	0.006	0.948

Data are expressed as means. ^a,b,c^ Values within the same row with different superscript letters indicate significant differences (*p* < 0.05). Abbreviations: BMD, bone mineral density.

**Table 7:** Effects of dietary 25-OH-VD_3_ combined with phytase and probiotics on the serum oxidative stress markers of weaned piglets.

Items	Treatments				SEM	*p*-Value
	CON	HI	CY	HICY		
T-AOC, mmol TE/L	0.10 ^c^	0.17 ^b^	0.19 ^a^	0.16 ^b^	0.021	0.031
CAT, U/mL	3.85	4.26	3.90	5.57	1.154	0.518
SOD, U/mL	21.8	22.5	23.1	22.7	0.476	0.251
MDA, nmol/mL	2.15	3.33	2.30	2.44	0.389	0.120
GSH-PX, mmol/L	1.41	1.34	1.22	0.88	0.123	0.152

Data are expressed as means (*n* = 6). ^a,b,c^ Values within the same row with different superscript letters indicate significant differences (*p* < 0.05). Abbreviations: T-AOC, Total Antioxidant Capacity; CAT, Catalase; SOD, Superoxide Dismutase; MDA, Malondialdehyde; GSH-PX, Glutathione Peroxidase.

**Table 8:** Effects of dietary 25-OH-VD_3_ combined with phytase and probiotic on the jejunal mucosal morphology of weaned piglets.

Items	Treatments				SEM	*p*-Value
	CON	HI	CY	HICY		
Villus height, μm	441.22	397.91	465.0	408.17	20.84	0.064
Crypt depth, μm	169.85	158.85	156.71	153.95	8.97	0.639
V/C	2.69	2.52	2.98	2.66	0.16	0.240

Data are expressed as means (*n* = 6).

## Data Availability

The original contributions presented in this study are included in the article/Supplementary Material. Further inquiries can be directed to the corresponding author.

## Supplementary Materials

The following supporting information can be downloaded at: https://www.mdpi.com/article/10.3390/nu18091428/s1, Table S1: The sequences of primer.

## Abbreviations

ADFI	Average feed intake
ADG	Average daily weight gain
AOAC	Association of Official Analytical Chemists
ASV	Amplicon sequence variant
BMD	Bone mineral density
CaBP	Calcium-binding protein
CaBP-D9k	Calcium-binding protein D9k
Calbindin-D28k	Calcium-binding protein D28k
CaSR	Calcium-sensing receptor
CYP27B1	Cytochrome P450 27B1
CP	Crude protein
DM	Dry matter
ECL	Chemiluminescence
ELISA	Enzyme-linked immunosorbent assay
F/G	Feed to gain ratio
GSH-PX	Glutathione peroxidase
HE	Hematoxylin-eosin
IKKA	IκB kinase α
IACUC	Institutional Animal Care and Use Committee
LDA	Linear discriminant analysis
MDA	Malondialdehyde
NRC	National Research Council
NF-κB	Nuclear factor-κB
OTUs	Operational taxonomic units
PCoA	Principal Coordinate Analysis
PVDF	Polyvinylidene difluoride
qPCR	Real-time quantitative PCR
SCFAs	Short-chain fatty acids
SLC34A1	Solute carrier family 34 (type II sodium/phosphate transporter), member 1
SDS-PAGE	Sodium dodecyl sulfate–polyacrylamide gel electrophoresis
SOD	Superoxide dismutase
TGF-β	Transforming Growth Factor-β
TRPV5/6	Transient receptor potential cation channel subfamily V member 5/6
VDR	Vitamin D receptor
25-OH-VD_3_	25-hydroxyvitamin D_3_
ZO-1	zonula occludens-1
