# Supplementary material for: Effects of 25-Hydroxyvitamin D3 Combined with Phytase and Probiotic on Calcium–Phosphorus Metabolism, Bone Development, and Growth Performance in Weaned Piglets
Source: Nutrients. 2026 Apr 30;18(9):1428. doi: 10.3390/nu18091428 (PMC13165107; doi:10.3390/nu18091428)
Supplement: Supplementary file 1 [file nutrients-18-01428-s001.zip › nutrients-4209133-supplementary.pdf]

Supplementary Table S1. The sequences of primer.

Gene	Primer sequence (5′→3′)	Length of PCR (bp)	Tm value (ºC)
β-actin	F: TGCGGCATCCACCAAACTA<br>R: CGTAGAGGTCCTTGCGGATGT	70	60
SLC34A1	F: CTATCTCCCCGCTCCCAGT<br>R: GAAGGCATAGGCTGAGGTCC	113	60
SLC34A2	F: TCGTGTGCTCCTTGGATGTG<br>R: CAACACGGAGAGCCAGTTGA	356	60
SLC34A3	F: GCCCCTCGCACTCACAC<br>R: CTTCCCACCCGCTCTTG	269	60
TRPV5	F: GCTCCCTTGAACCACATCCCT<br>R: GTGGGCTTCATCCAGTTGCT	162	60
TRPV6	F: GCTGTGGTCATCCTGGGCTTTG<br>R: AATGATGGCGAAGGCGGCATAG	204	60
CYP27B1	F: ACACAGAGACCTTCATCCGC<br>R: GTGTCCACTCCAGCCAGTAG	313	60
VDR	F: TGGTTGGAAGTGTCTGGGAG<br>R: GGGGTCAGGTAAGGAAGTGC	117	60
CaSR	F: ACTGAGCCCTTTGGGATTG<br>R: TTGACGATGGGCGTGTTT	107	60
CaBP-D9k	F: AGAGCAAATGCACCTCTTGG<br>R: CATGTGAGCGCATAGAAGGA	105	60
CaBP-D28k	F: TATGCAGCCAAAGAAGGGGAT<br>R: CTAGGGTTCTCGGACCTTTCAG	103	60
Claudin-1	F: AAGATTTACTCCTACGCTGGT<br>R: CTTGGTGTTGGGTAAGATG	500	60
ZO-1	F: GGATGGTCACACCGTG<br>R: GGAGGATGCTGTTGTCTC	167	60
Occludin	F: ACGAGCTGGAGGAAGACTGGATC<br>R: TGAGCCGTACATAGATCCAGAAGC	235	60
TLR4	F: CCGTCATTAGTGCGTCAGTTCT<br>R: TTGCAGCCCACAAAAAGCA	100	60

NF-κB	F: AACCCCTTCCAAGTTCCCA<br>R: TCCCCGAGTTCCGATTCAC	199	60
IKKA	F: AATCTGCTTCGGAACAACA<br>R: GTCAATCTGGATGCTGGTT	111	60

Abbreviations: SLC34A1/ SLC34A2/ SLC34A3, solute carrier family 34 (type II sodium/phosphate transporter), member 1/ member 2/ member 3; TRPV5/ TRPV 6, transient receptor potential cation channel subfamily V member 5/ member 6; CYP27B1, cytochrome P450 27B1; VDR, vitamin D receptor; CaSR, calcium sensing receptor; CaBP-D9k, calcium-binding protein D9k; CaBP-D28k, calcium-binding protein D28k, ZO-1, zonula occludens - 1; TLR-4,toll-like receptor 4; NF-κB, nuclear factor-κB; IKKA, IκB kinase α. . All genes were normalized using the expression level of β-actin.
